# Extracorporal membrane oxygenation for cardiopulmonary support after open heart surgery

**DOI:** 10.1186/cc9431

**Published:** 2011-03-11

**Authors:** UJ Jaschinski, G Kierschke, H Forst, M Beyer

**Affiliations:** 1 Klinikum Augsburg, Germany

## Introduction

Arterial-venous extracorporal membrane oxygenation (ECMO) is a rescue tool in acute heart failure after cardiopulmonary bypass (CPB) when separation from CPB cannot be achieved by conventional means (volume, inotropes, intra-aortic counterpulsation IABP). The role of ECMO in this scenario is far from clear and factors predicting a poor outcome are lacking. However, such indices would be helpful to find a reasonable approach.

## Methods

Analysis of a prospective evaluated dataset in a surgical ICU of a university teaching hospital.

## Results

In 19 patients (mean age 58 years) with postcardiotomy cardiogenic shock despite high-dose medication with inotropes and normal filling pressures, separation from CPB was not possible. These patients were scheduled for ECMO. The mean preoperative EF was 20.8% and in 47.3% of the patients cardiopulmonary resuscitation (CPR) had to be performed already before CPB. Eleven patients (57.8%) received an IABP before ECMO. The most frequent complications in the ICU were: arrhythmia (63.1%), bleeding (78.9%), renal failure with CRRT (47.3%) and respiratory failure (paO_2_/FiO_2 _< 250 mmHg) (100%). The mean duration on ECMO was 6.8 days, mean stay in the ICU was 13.1 days and mean hospital stay was 44.5 days. Only 6/19 patients survived (31.5%) and were discharged from hospital. These patients except one had no CPR in the preoperative period.

## Conclusions

ECMO in acute heart failure after adult open heart surgery in this series had an enormous high mortality of 68.5%. However, these results are in line with other series with a reported mortality of 67 to 75.2% [1,2]. CPR in the preoperative setting seems to be a grave sign for survival and in these patients ECMO is not recommended since mortality reaches an unacceptable high rate. This statement needs to be confirmed by an adequate powered trial.
